# A machine learning method based on the genetic and world competitive contests algorithms for selecting genes or features in biological applications

**DOI:** 10.1038/s41598-021-82796-y

**Published:** 2021-02-08

**Authors:** Yosef Masoudi-Sobhanzadeh, Habib Motieghader, Yadollah Omidi, Ali Masoudi-Nejad

**Affiliations:** 1 Research Center for Pharmaceutical Nanotechnology, Biomedicine Institute, Tabriz University of Medical Sciences, Tabriz, Iran; 2 Department of Bioinformatics, Biotechnology Research Center, Tabriz Branch, Islamic Azad University, Tabriz, Iran; 3 Department of Basic Sciences, Gowgan Educational Center, Tabriz Branch, Islamic Azad University, Tabriz, Iran; 4 Department of Pharmaceutical Sciences, College of Pharmacy, Nova Southeastern University, Fort Lauderdale, Florida, 33328 USA; 5 Laboratory of Systems Biology and Bioinformatics (LBB), Institute of Biochemistry and Biophysics, University of Tehran, Tehran, Iran

**Keywords:** Classification and taxonomy, Computational models, Data mining, Machine learning

## Abstract

Gene/feature selection is an essential preprocessing step for creating models using machine learning techniques. It also plays a critical role in different biological applications such as the identification of biomarkers. Although many feature/gene selection algorithms and methods have been introduced, they may suffer from problems such as parameter tuning or low level of performance. To tackle such limitations, in this study, a universal wrapper approach is introduced based on our introduced optimization algorithm and the genetic algorithm (GA). In the proposed approach, candidate solutions have variable lengths, and a support vector machine scores them. To show the usefulness of the method, thirteen classification and regression-based datasets with different properties were chosen from various biological scopes, including drug discovery, cancer diagnostics, clinical applications, etc. Our findings confirmed that the proposed method outperforms most of the other currently used approaches and can also free the users from difficulties related to the tuning of various parameters. As a result, users may optimize their biological applications such as obtaining a biomarker diagnostic kit with the minimum number of genes and maximum separability power.

## Introduction

In computational biology, researchers may be involved with the handling of large omics datasets with many features (e.g., genomics, proteomics, metabolomics, etc.)^1^. For instance, the total number of profiled genes is usually more than 20,000 in human samples, which have been exploited for different purposes such as the detection of biomarkers^2^. Given that the number of features from proteomics and metabolomics data is potentially much larger^3^, it is almost impossible to extract a set of biomarkers kit of a manageable size from such large data sets^4^. For instance, in the field of genomic data, researchers aim to (i) select genes having higher separability power between different states, such as cancerous and noncancerous samples, and (ii), confine them to a reasonable number to be handled^5^. From the machine learning perspective, features or genes can be categorized into three classes as follows:

(i) Negative features^6^, which can mislead a learner and reduce its performance. Thus, they must not be selected in the application.
(ii) Neutral features^7^, which do not play any role in the performance of a learner and can only increase the time of predicting. Like the first group, these features should be avoided.
(iii) Positive features^8^, which play a determinant role in distinguishing between samples and enhance the performance of a learner. For such features, the feature selection (FS) methods need to be applied since some of the features may have redundant roles as others. Further, a large set of them may be represented by a small set.

Due to the combinatorial nature of FS, it is a nondeterministic polynomial (NP-hard) problem that cannot be solved in a polynomial-time order, in large part because of being accepted by nondeterministic Turing machines^9^. To overcome the time complexity, heuristic and metaheuristic algorithms, which find acceptable answers to these problems, have been developed^10^.

In different studies, it has been shown that the metaheuristic algorithms, which do not confine themselves to a specific range of the search space, are generally more suitable than heuristic algorithms^11–13^. In addition, two-step methods may obtain better results than single methods^14,15^. Therefore, in this study, we capitalized on a two-step method, which is based on a genetic algorithm (GA)^16^ and our previously developed world competitive contests (WCC) optimization algorithm^17^, the so-called “GA_WCC method”. In the first step of the GA_WCC method, the GA reduces the total number of features to a minimum upper bound. Next, the WCC selects an optimal subset of features for the desired application. Overall, the GA_WCC method is based on a two-step process for FS, which (i) does not require limiting the number of features to a predefined value, and (ii) outperforms other currently used methods.

## Related works

In this section, we discuss the limitation of related approaches works that can be divided into six classes as follows:

(i) Filter methods: These techniques look for the relationships among features and investigate how much information exists in a feature. For this purpose, various mathematical formulas have been proposed, including Entropy^18^, mutual information^19^, Fisher score^20^, correlation^21^, Laplacian^22^, etc. Although these approaches are simple and have a low time-complexity, their performance is lower than the other categories^23^. To tackle such a limitation, wrapper-based method has been developed and are built-upon in this paper.
(ii) Wrapper methods: Unlike the first class, these approaches score the selected features by a learner such as a support vector machine (SVM)^24^, artificial neural networks (ANN)^25^, decision tree (DT)^26^, or others^27–29^. Usually, optimization algorithms are applied to select an optimal subset of features^30,31^. In different studies, it has been shown that these approaches can achieve remarkable outcomes^32^, but most of the FS studies do not employ state-of-the-art algorithms for the FS. Here, we used the WCC algorithm for the FS problem.
(iii) Ensemble methods: For the FS, ensemble methods create a learner such as a decision tree^33^ and selects features in such a way that the learner chooses them for generating a model^34,35^. Due to their greedy nature, ensemble methods may fall into local optima solutions and do not reach the optimal result. To deal with this limitation, we introduce the WCC algorithm, which features a low probability of falling into local optima.
(iv) Hybrid methods: A combination of the three mentioned methods is applied to the FS problem^36^. For example, the total number of features is reduced by filter methods, and then an optimal subset of features is chosen by wrapper or ensemble methods^37,38^. In this class of related works, it is essential to combine the algorithms properly. Therefore, we assumed that a combination of wrapper-wrapper approaches, which merge two wrapper-based algorithms, might be a suitable option for FS.
(v) Hypothesis-based studies: A concept is hypothesized based on prior knowledge and the correctness of which is tested via various experiments on gold-standard datasets^39^. Although these techniques can help in making a proper decision, they do not prevent the mentioned limitations.
vi) Review works: These works survey different methods such as filter^40^, wrapper^41^, ensemble^42^, hybrid^43^, and discuss their advantages and disadvantages. Further, they study the role of FS in diverse areas and often constitute the future directions^44^.

## Materials and methods

### The datasets

Several datasets with diverse properties have been selected from various sources such as the machine learning repository developed at the University of California Irvine (UCI)^45^ and published seminar literature sources. For every dataset, the total number of samples is almost the same in its different classes. Table 1 shows the properties of the datasets and describes them.

**Table 1:** The properties of the datasets.

Dataset’s name	Description	Number of samples	Number of features	Type	Number of classes	Missing value	References
Arrhythmia	Cardiac arrhythmia	452	279	Classification	16	Yes	^45^
Cancer	Adeno carcinoma	499	17,995	Classification	2	Yes	^46^
CHD	Cardiovascular heart diseases	303	45	Classification	5	No	^47^
Diabetes	Diabetes hospital between 1999 and 2008	100 000	55	Classification and clustering	2	No	^47^
Drug	QSAR information between molecular properties and IC_50_	56	223	Regression	–-	No	^36^
PID	Pima Indian diabetes	768	9	Classification	2	No	^48^
SHD	Statlog heart data	270	13	Classification	2	No	^48^
WDBC	Wisconsin Diagnostic Breast Cancer	198	34	Regression	–	Yes	^48^
Lung	Lung cancer	32	56	Classification	2	Yes	^48^
QSAR	Molecular fingerprints correspond to androgen receptor	1 687	1025	Classification	2	No	^49^
Arcene	Mass-spectrometric data correspond to cancer and normal patients	900	10,000	Classification	2	No	^50^
MicroMass	Microorganism identification from mass-spectrometry data	931	1300	Classification	2	No	^51^
RNA-Seq	Gene expression of patients	801	20,531	Classification	5	No	^52^

### The proposed method

**Figure 1:**
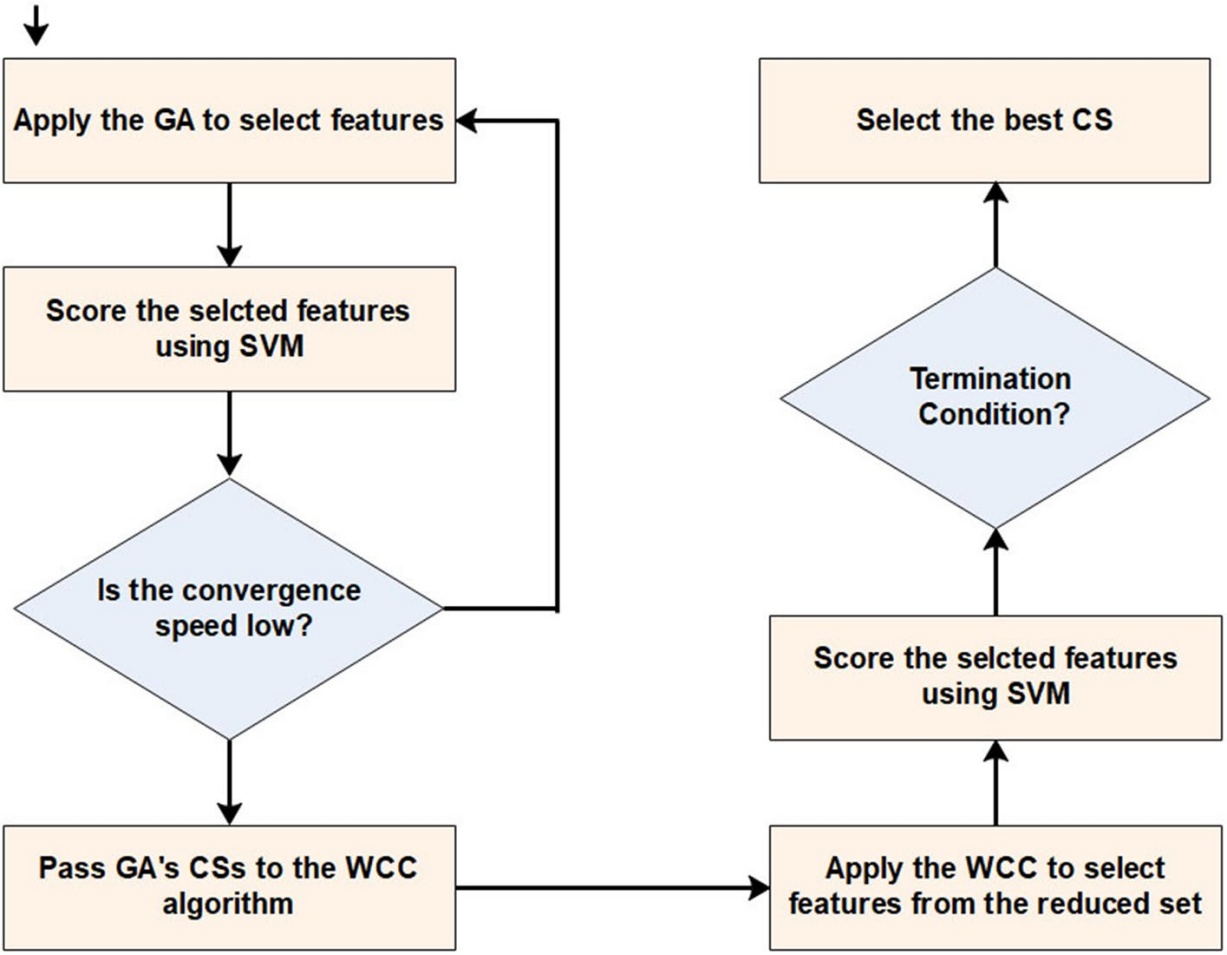
The framework of the proposed method based on the wrapper-wrapper feature selection technique, consisting of two phases. First, the GA confines the total number of features and then passes them towards the WCC. All the CSs are scored by the SVM. At the end of the method, the best CS is introduced as an answer to the problem. CS, candidate solution; GA, genetic algorithm; SVM, support vector machines; WCC, world competitive contests algorithms.

Our proposed GA_WCC method (Fig. 1) selects the features using a two-step wrapper approach. To this end, as the first step, the Genetic Algorithm (GA) limits the total number of genes or, generally, features, and then the World Competitive Contests (WCC) selects an optimal subset of them from the reduced set of features. Overall, this study has been established based on the following rationale:

(i) The GA starts with a first population of candidate solutions, which each consists of several variables (a subset of features). Unlike other optimization algorithms such as the particle swarm optimization (PSO)^53^, for the GA, the probability of falling into local optima is minimal, because it produces a high number of candidate sets. However, the convergence speed of GA is usually less than other optimization algorithms (e.g., TLBO^54^ and FOA^55^). Hence, this limitation may be addressed when the GA algorithm is combined with other state-of-the-art optimization algorithms. This issue is considered in the present study, by merging the GA and WCC algorithm.
(ii) The WCC begins with a first population of potential answers and applies its all the operators to all the existing candidate solutions (CSs), so it spends more times than other optimization algorithms. Hence, when applying the WCC algorithm to an optimization problem, the total number of CSs is limited. This algorithm has an acceptable convergence speed, but the main limitation of WCC relates to its complex stages, which increase the execution time. Further, for a CS, WCC calls the cost function more than other algorithms due to the nature of its operators. At the last steps of the algorithm, the applied operators make CSs similar to each other, so the convergence speed of the algorithm is reduced (due to the limited number of CSs).

**Figure 2:**
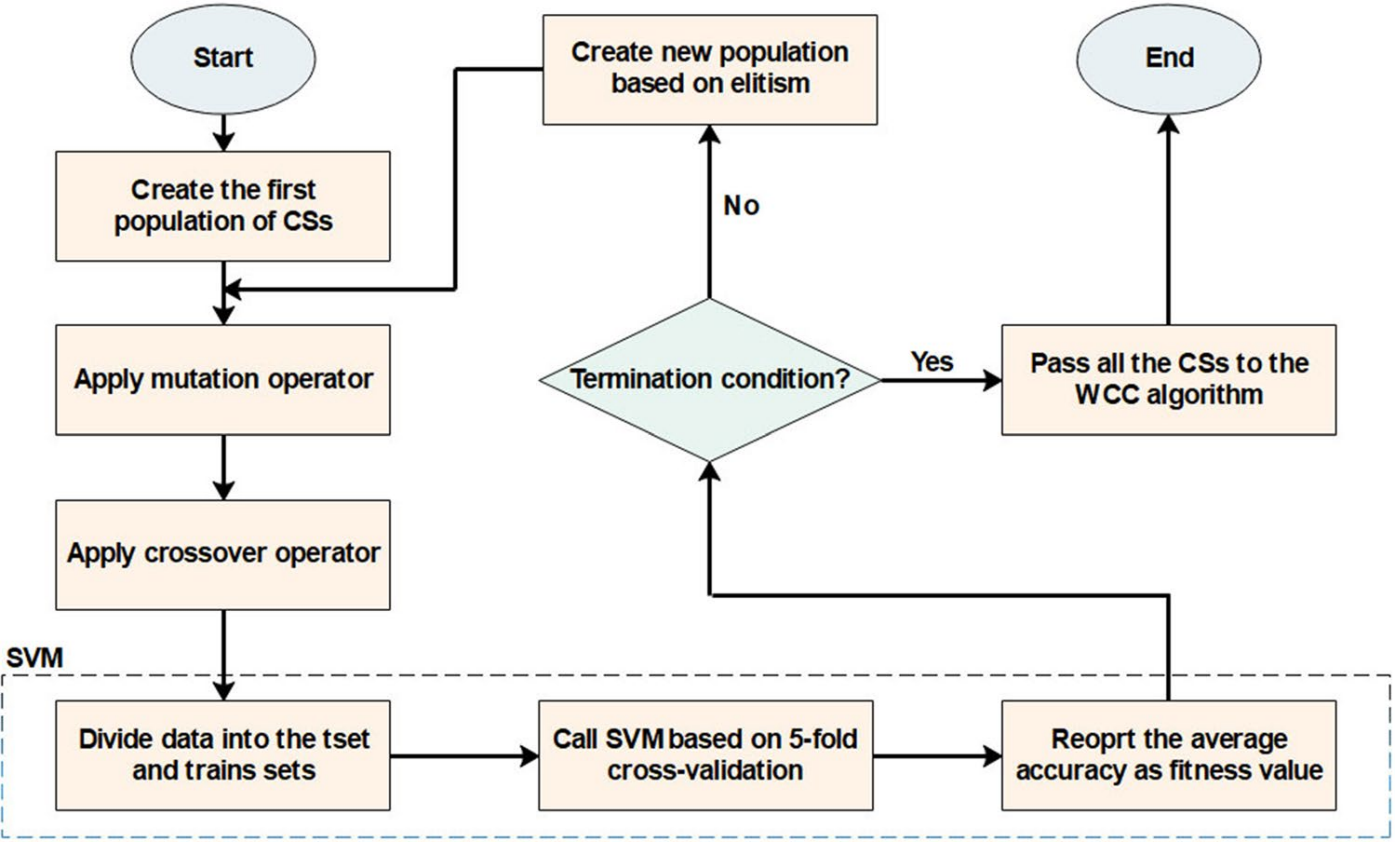
Flowchart of the employed GA. This algorithm begins with several randomly generated potential answers (a subset of the existing features) and applies its operators to produce new CSs, which contain the selected features. To calculate fitness of the CSs, a model is created using SVM, and its accuracy (based on fivefold cross-validation) is reported. Also, to generate new population, elitism method (which generates new population based on the CSs having the higher value of fitness) has been used. CS, candidate solution; GA, genetic algorithm; SVM, support vector machines.

**Figure 3:**
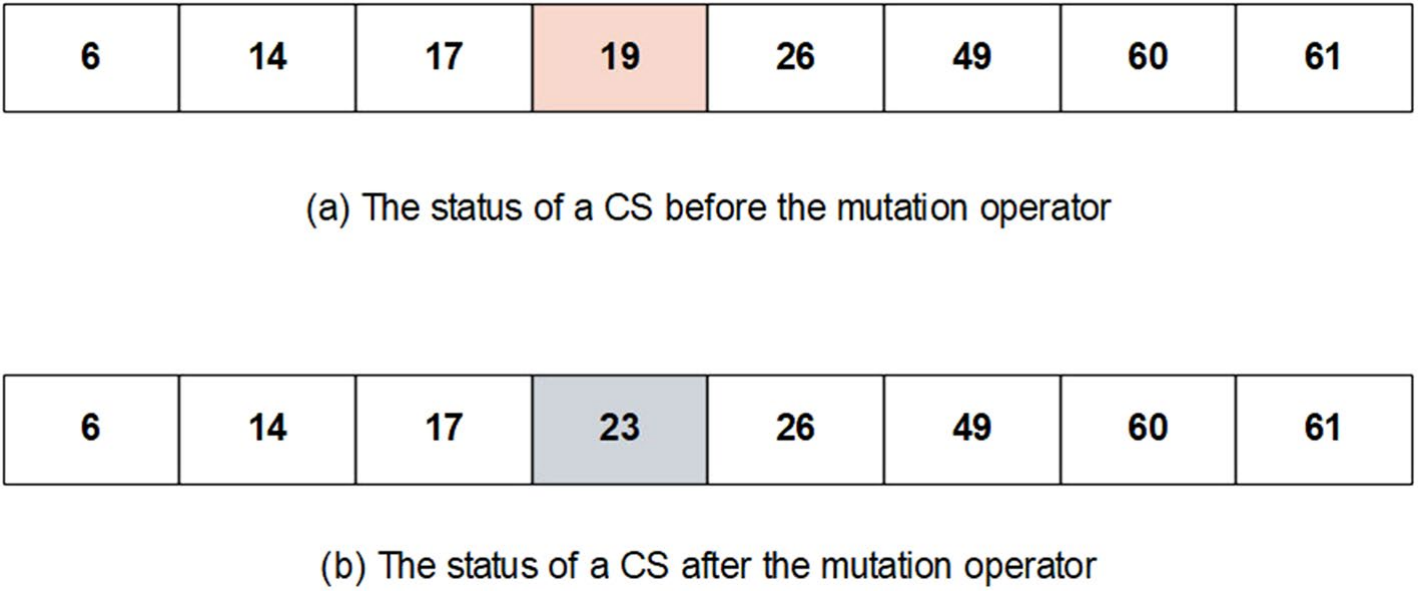
An example of the utilized mutation operator, which choses a variable randomly and changes its value. (**a**) The status of a CS has been shown before applying the operator. (**b**) The status of a CS has been shown after applying the operator. CS, candidate solution.

**Figure 4:**
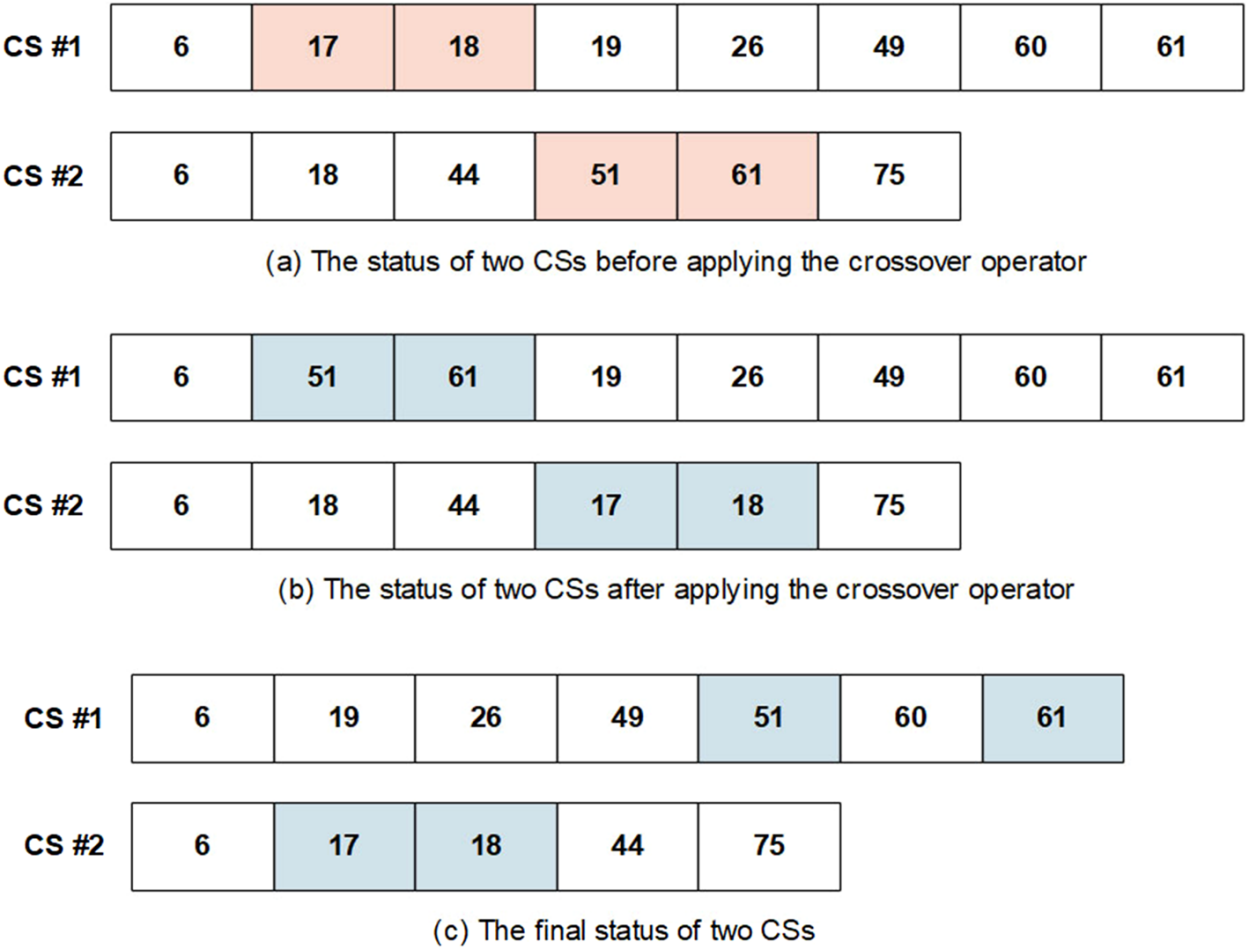
An instance of the utilized crossover operator. (**a**) A range from two CSs are randomly chosen, which their length is the same. (**b**) The values of the specified ranges are transferred. (**c**) After changing the values, the repeated values are removed, and the others are sorted. CS, candidate solution.

**Figure 5:**
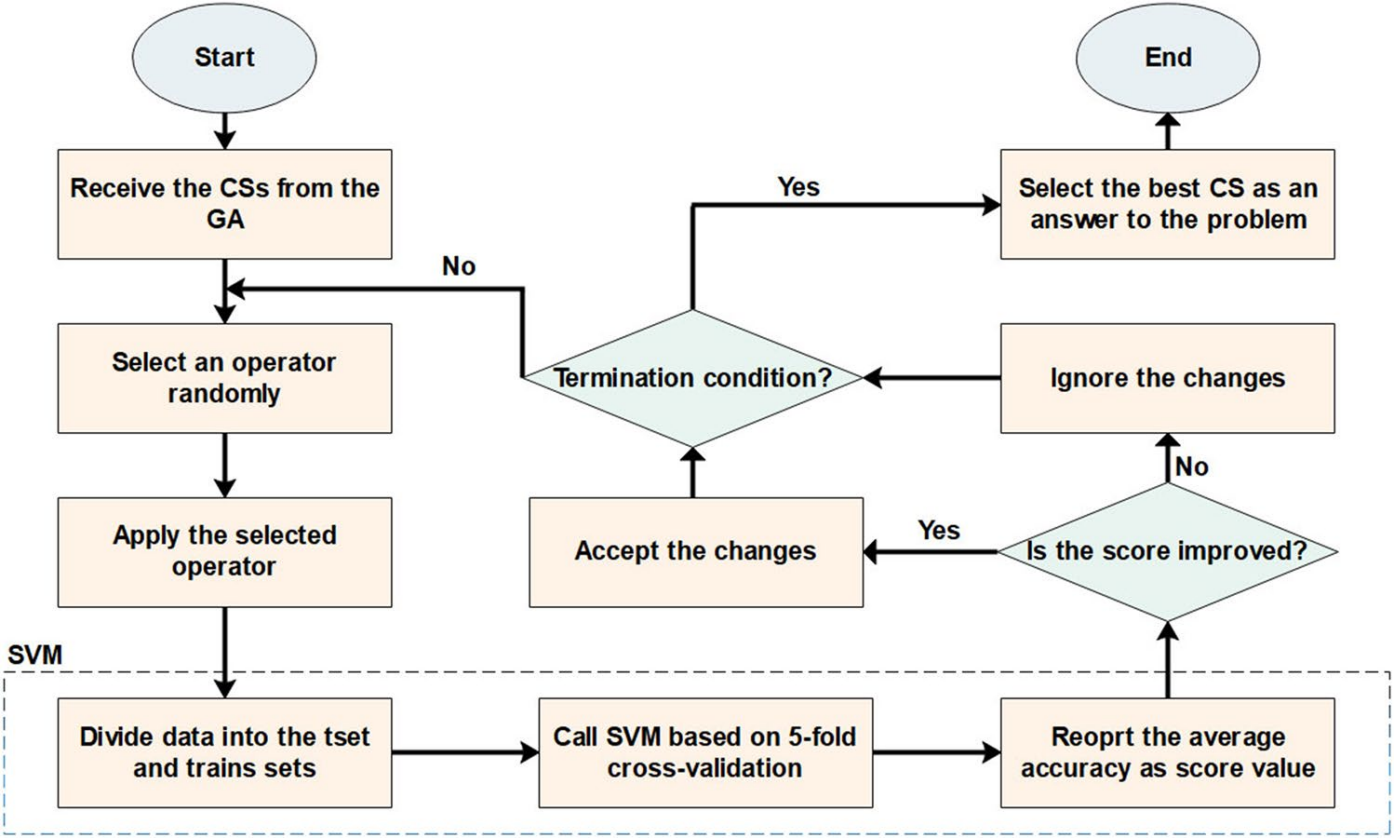
Flowchart of the WCC algorithm (the algorithm developed here). The WCC algorithm receives its first population of CSs from the GA and applies its operators on them. If the changes induced by the operators improve the accuracy, they are accepted. Otherwise, the changes are ignored. CS, candidate solution; GA, genetic algorithm; SVM, support vector machines.

**Figure 6:**
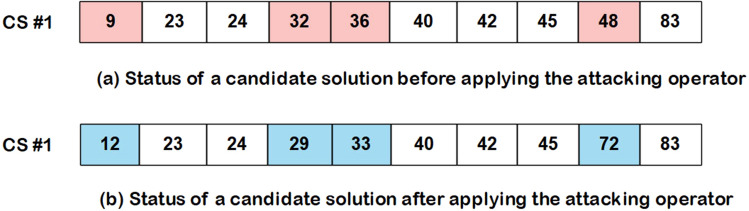
An example of the attacking operator. The array presents a CS which includes a set of the features. (**a**) Some indices of a CS are randomly chosen. The values of the selected indices have been highlighted by the pink colors. (**b**) The values of the determined indices are replaced by other randomly generated values. The blue colors show the replaced values. If repeated values appear, one of them remains and others are eliminated. The size of a CS may be reduced after applying the attacking operator. CS, candidate solution.

**Figure 7:**
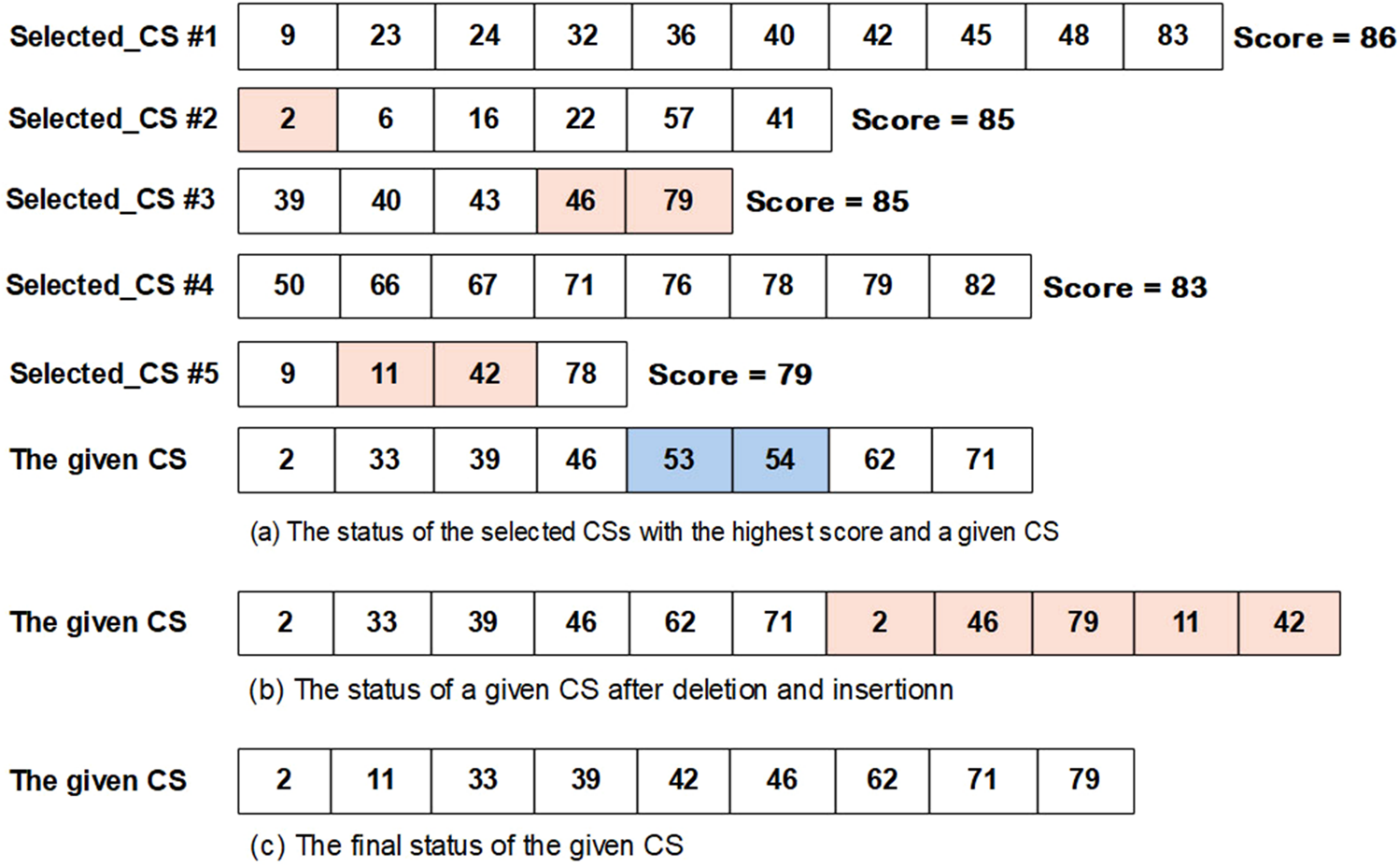
An instance of the transferring operator. The arrays present CSs which include a set of different features. (**a**) Five CSs with the highest scores are selected. The colored indices are the variables (features), which are imported to the given CS. The blue colors are the variables that are randomly selected and are removed from the given CS. (**b**) The status of the given CS is shown after deleting the blue highlighted features and inserting the pink highlighted features. (**c**) The finalized status of the given CS is presented. In this step, the selected features are sorted, and the repeated ones are removed. CS, candidate solution.

**Figure 8:**
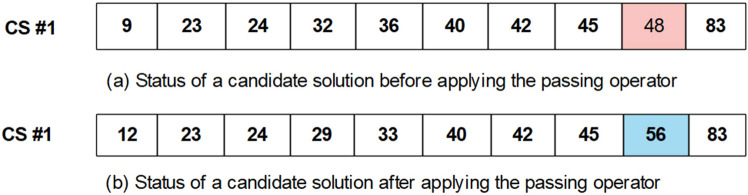
An example of the passing operator. The array presents a CS which includes a set of the features (**a**) The status of a CS before applying the passing operator is presented. One of the features has been randomly selected. (**b**) The status of the CS after applying the passing operator. The determined feature has been replaced by another feature. CS, candidate solution.

Optimization algorithms differ from each other from a way that they change CSs (the operators of the algorithms). In this study, the WCC algorithm is developed to the FS problem, and its operators are modified to select an optimal subset of features. Given the advantages and disadvantages of the GA and WCC algorithm (the modified version of the WCC algorithm), it is expected that their limitations will be diminished when combined with each other. Inspired by this idea, this study has been designed, and an efficient two step feature selection method based on a wrapper approach has been introduced. As shown in Fig. 1, the GA_WCC method includes several steps as follows:

(i) Applying the genetic algorithm: In the first step of the proposed method, a version of GA is used for the FS^56^. In different FS studies, CSs are binary, while their length is constant and equal to the total number of features. In this study, for both GA and WCC algorithms, CSs have variable sizes and contain the indices of the selected features. In the optimization scope, the GA is the basis for other optimization algorithms. However, GA generally exhibits a low level of performance in comparison with other algorithms. This notwithstanding, GA produces different CSs, which may help other optimization algorithms to obtain better results^57^. In Fig. 2, the flowchart of the employed GA is shown, which includes the following main steps:
  - Creating a first population of CSs: potential answers or CSs are called ‘chromosomes’ in the GA algorithm, and their values of genes are randomly quantified. Every CS incorporates some features, which are chosen from a given feature set (the total number of variables in a CS depends on the size of a dataset). In the proposed method, initially, the CSs have an identical length, but their length may vary from each other because of some repeated values. For instance, in generating initial CSs, it is possible that a CS contains some repeated features. In such a case, only one of the repeated values is remained and the remaining ones are ignored.
  - Applying GA operators: The GA consists of three main operators named mutation, crossover, and selection. In the employed mutation operator, a variable of a chromosome is randomly selected, and its value is replaced by another randomly selected variable. In the crossover operator, two ranges of the CSs with the same length are randomly chosen, and their contents are exchanged. Finally, in the selection operator, elitism technique has been used, which forms the new population based on the most deserve chromosomes of the current population. In Figs. 3 and 4, the instances of the mutation and crossover operators are depicted, which describes, how the mentioned operators are applied to generating new CSs.
  - Scoring the selected features: The proposed method is a wrapper method in which a learner evaluates the selected features. Due to the nature of the datasets, which are approximately class-balanced, we basically use the accuracy score (Eq. 1). Other criteria are also inspected in the experimental section.
    1 $$Score = Accuracy = \frac{TP + TN}{{TP + TN + FP + FN}}$$
    where TP, TN, FP, and FN represent true positives, true negatives, false positives, and false negatives, respectively. Because of their performance and reasonable time in generating a model, Support Vector Machines (SVMs) have been used for assessing the CSs. Considering the popularity and performance of SVMs, many libraries and packages have been developed accordingly. In this study, the LibSVM library, which is one of the most popular libraries with different options, was employed^58^.
  - Investigating the termination condition: when the value of the best CS is remained constant for 10 consequent iterations (generations), the GA is terminated, and all its CSs are passes to the WCC algorithm.
(ii) Applying the proposed algorithm (the WCC): As mentioned before, at the end of the first step, GA passes the created CSs to the proposed algorithm (the flowchart of the WCC algorithm is shown in Fig. 5) and constitutes its first population of CSs. Next, WCC changes the CSs using its operators, which are explained and formulated as follows:
  - Attacking operator: For a given CS, this operator selects some variables randomly and assigns them new values by chance from [1, n], where n is the total number of the existing features/genes. Equation 2 formulates the attacking operator:
    2 $$\mathop \sum \limits_{i = 1}^{k} \left[ {CS\left( r \right) = rand\left( n \right)} \right]$$
    where CS, n, and k are a given candidate solution, the total number of features, and an integer random value between 1 and n, respectively. In other words, the k parameter determines how many variables of a CS must be changed. Further, the sigma sign denotes a loop, and r is an integer value between 1 and n as is k. Here, is an example of the attacking operator in Fig. 6.
  - Transferring operator: Based on the scores (classification accuracy using a given CS), this operator selects several CSs with the highest score (*Selected_CS*), and then, chooses randomly some values (features) from them. Next, for a given CS, this operator imports the selected values. Equation 3 formulates the mentioned steps. Figure 7 describes the transferring operator in detail.
    3 $$\mathop \sum \limits_{j = 1}^{R} \mathop \sum \limits_{i = 1}^{k} \left[ {CS\left( r \right) = selected_{CSm} \left( {rand\left( l \right)} \right)} \right]$$
    where $l$, R, and m are the length of the *selected_CS*, a random integer value between 1 and the total number of selected CSs, and an index which shows the randomly *selected_CS*, respectively. Further, other parameters have been described in Eq. 2.
  - Passing operator: While the transferring and attacking operators may result in large changes in a CS, this operator guarantees low modifications. For this purpose, the operator only selects a variable by chance and changes its value. Equation 4, whose parameters are explained in Eq. 2, formulates the passing operator.
    4 $$CS\left( r \right) = rand\left( n \right)$$
    Figure 8 illustrates an example of the passing operator and explains how the operator can be applied to the FS problem.
    Each of the changes induced by the operators will be accepted if they increase the accuracy score. Further, repeated features may appear by applying the operators. In these situations, only one of the repeated features is kept and all others are removed. Hence, the length of CSs may vary.
(d) Investigating the termination conditions: For terminating the algorithms, several options (e.g., predefined number of iterations, time, accuracy, etc.) can be used. In the present study, two different strategies are chosen for terminating the algorithm. As mentioned before, when the value of accuracy remains about constant in the last ten iterations, the GA is finished. For the WCC algorithm, a predetermined number of iterations has been considered as the termination condition.

## Results

**Figure 9:**
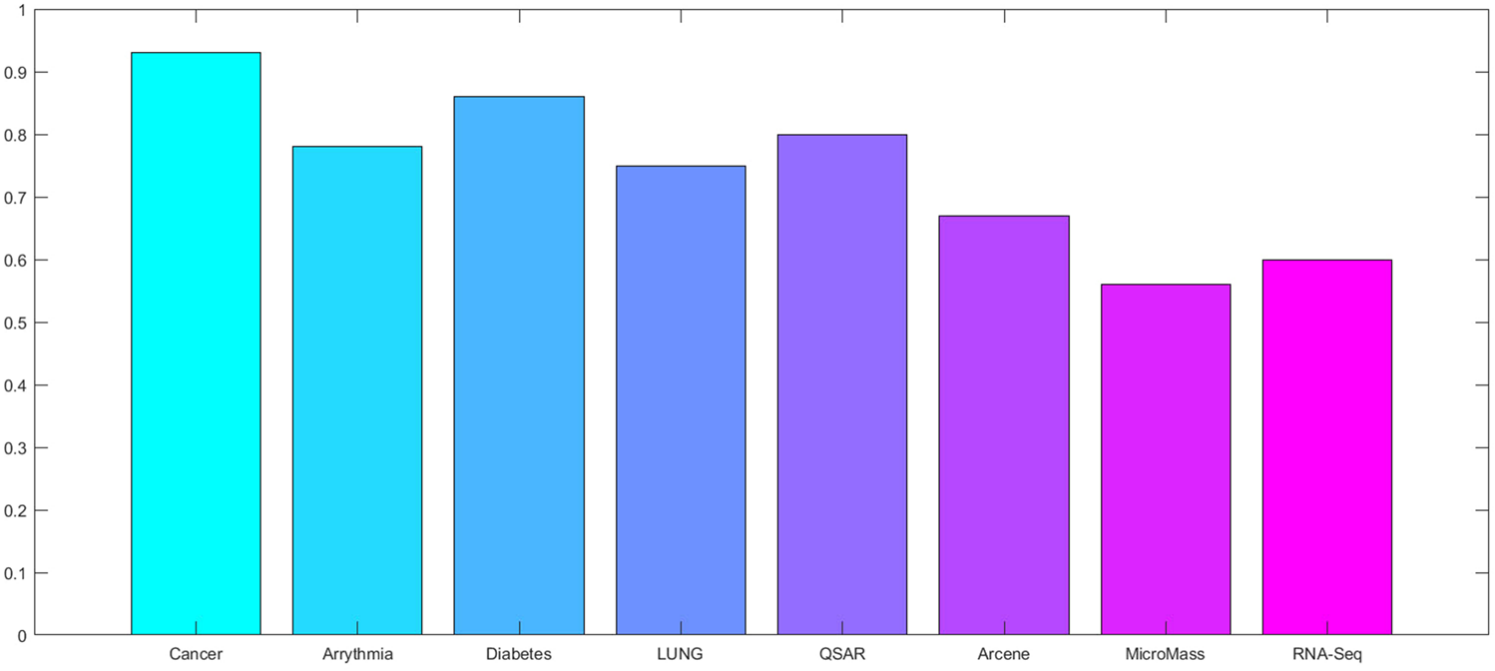
The performance of the SVM without applying FS algorithms on the datasets.

**Figure 10:**
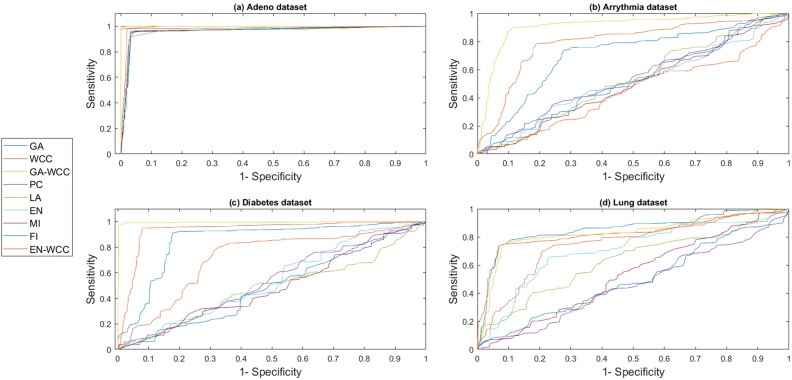
ROC curves of the methods on the first category of datasets. (**a**) The ROC curve of the manners on the Cancer dataset (Adeno dataset). Although all the methods have acceptable performance, the proposed method (GA_WCC) has higher separating power relative to the others. (**b**) The ROC curve of the methods on the Arrhythmia dataset. Like the first section, GA_WCC show better performance in term of classifying power. (**c**) The ROC curve of the manners on the Diabetes dataset. These results also validate that two-step and wrapper methods obtain better results than filter FS methods. (**d**) The ROC curve of the manners on the Lung dataset. In addition to accrediting the results of the three mentioned section, these diagrams state that EN reaches to a better solution than other filter approaches, and its combination with WCC improves the performance of a model. ROC, receiving operating characteristic; WCC, world competitive contests algorithm; GA, genetic algorithm; PC, Pearson correlation; LA, Laplacian score; EN, entropy; MI, mutual information; FI, Fisher score.

**Figure 11:**
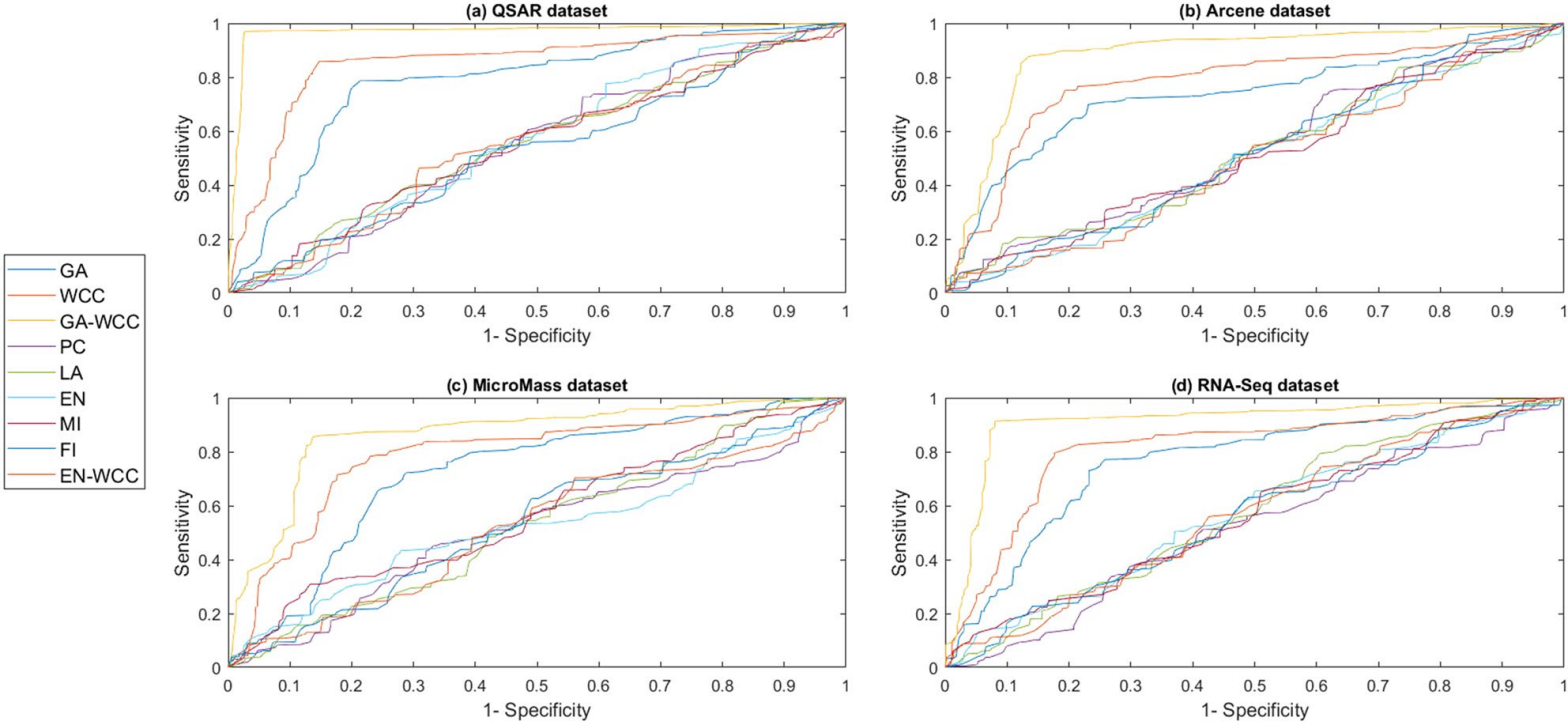
ROC curves of the methods on the second category of datasets (high dimensional). (**a**) The ROC curve of the manners on the QSAR dataset. (**b**) The ROC curve of the methods on the Arcene dataset. (**c**) The ROC curve of the methods on the MicroMass dataset. (**d**) The ROC curve of the manners on the RNA-Seq dataset. Like Fig. 10, these diagrams state that optimization algorithm can acquire better results than other algorithms, and two-step feature selection method may be a suitable technique for choosing the most effective features or genes in the biology field. ROC, receiving operating characteristic; WCC, world competitive contests algorithm; GA, genetic algorithm; PC, Pearson correlation; LA, Laplacian score; EN, entropy; MI, mutual information; FI, Fisher score.

**Figure 12:**
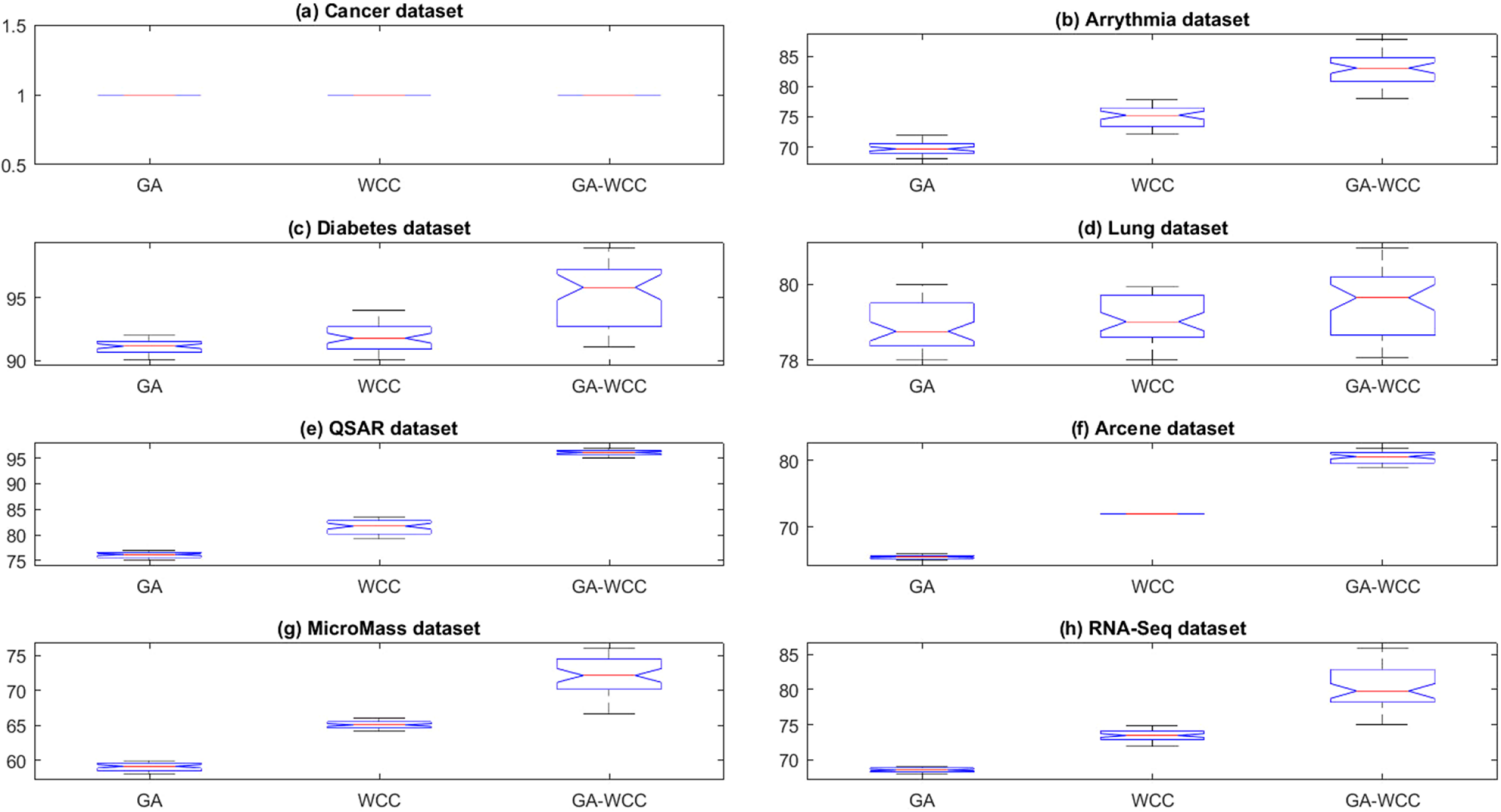
Boxplot of the GA, WCC, GA_WCC algorithms on the (**a**) Cancer, (**b**) Arrythmia, (**c**) Diabetes, (**d**) Lung, (**e**) QSAR, (**f**) Arcene, (**g**) MicroMass, and (**h**) RNA-Seq datasets. For the Cancer dataset, all the algorithms have shown an identical performance. For the remaining ones, although the error value of the GA and WCC algorithms is lower than the GA_WCC method, the proposed method has outperformed the GA and WCC algorithms in terms of accuracy and other classification criteria. WCC, world competitive contests algorithm; GA, genetic algorithm;

**Figure 13:**
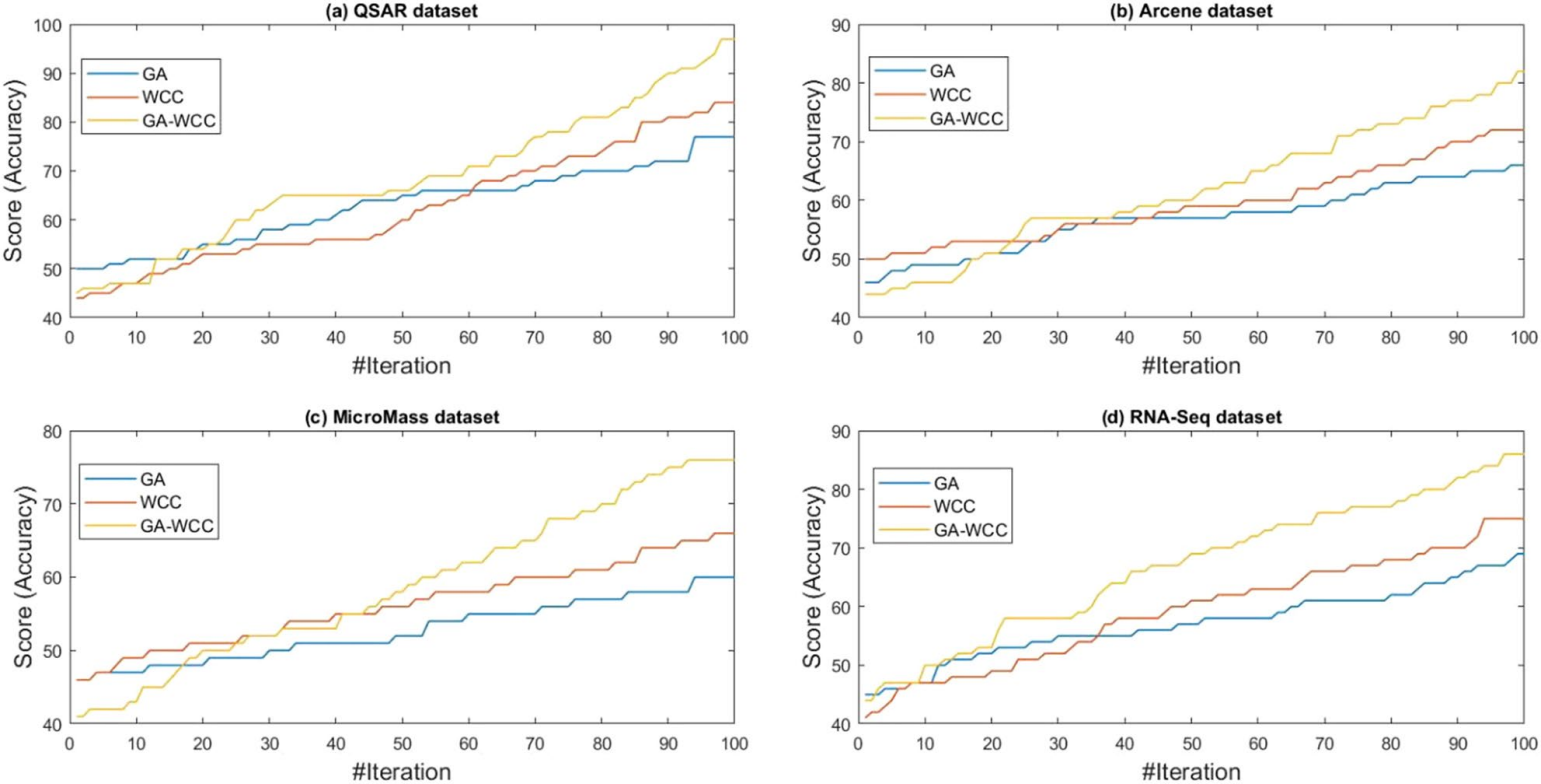
Convergence of the GA, WCC, and GA-WCC algorithms on the (**a**) QSAR, (**b**) Arcene, (**c**) MicroMass, and (**d**) RNA-Seq datasets. In the early stages of the iterations, the algorithms present a similar behavior. However, after elapsing more times, the WCC algorithm shows better performance than the GA, and the proposed method exhibits proper convergence behavior than the WCC algorithm.

**Figure 14:**
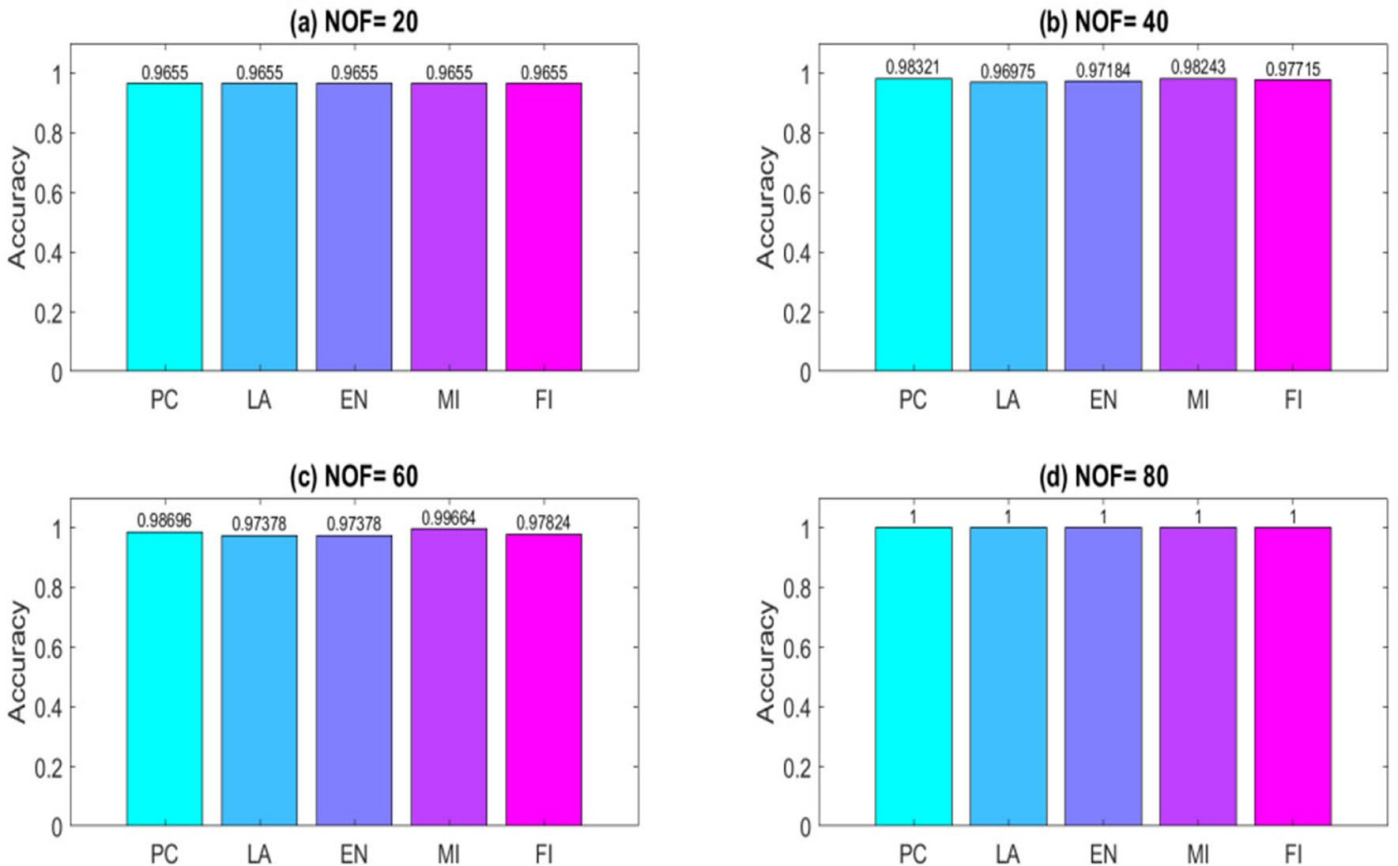
Outcomes of the filter FS methods on the Cancer dataset with selecting (**a**) 20, (**b**) 40, (**c**) 60, and (**d**) 80 features. When the number of features (NOF) is confined to 80, all the ways acquire the best possible solution. PC, Pearson correlation; LA, Laplacian score; EN, entropy; MI, mutual information; FI, Fisher score.

**Figure 15:**
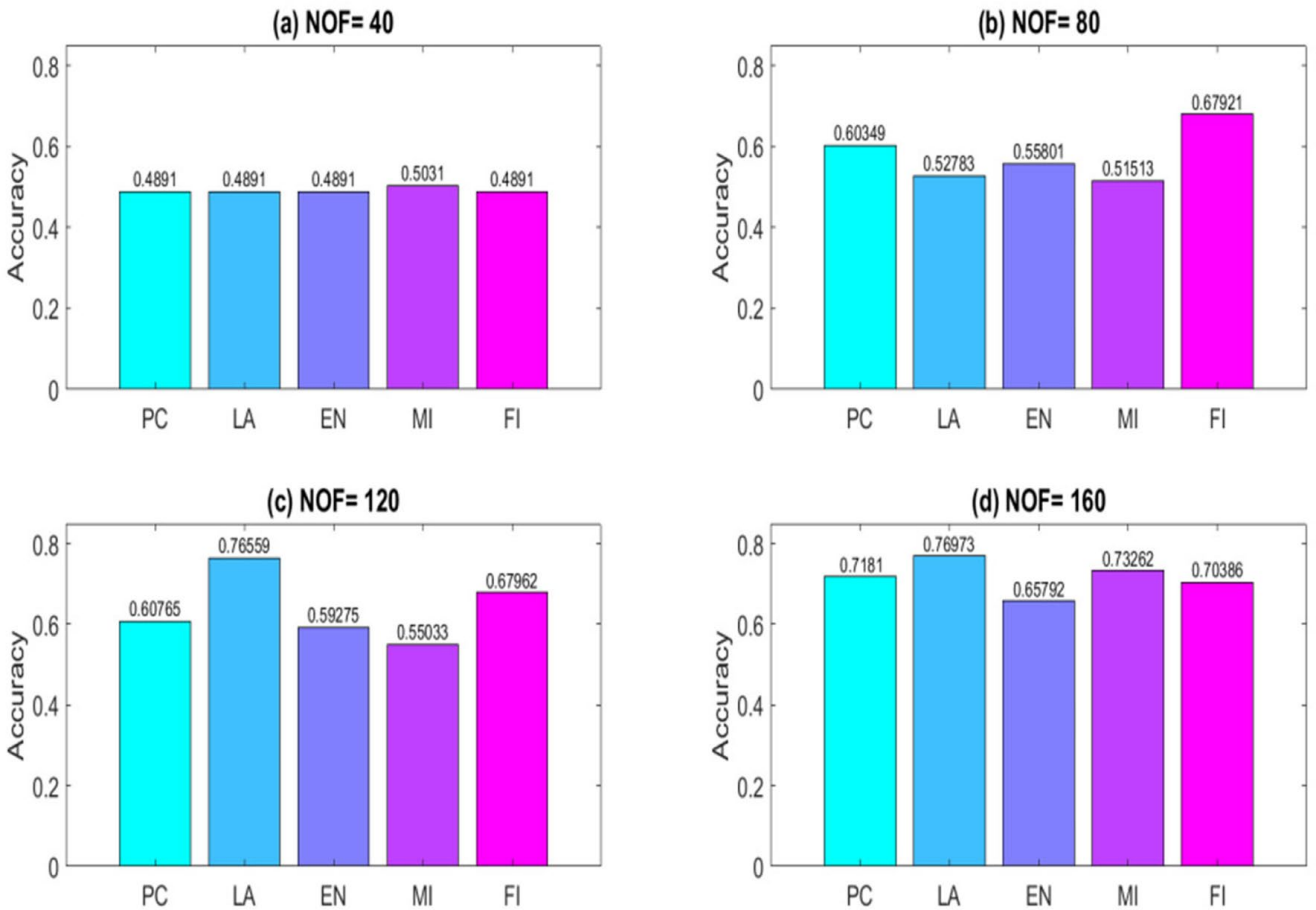
Outcomes of the filter FS methods on the Arrhythmia dataset with selecting (**a**) 400, (**b**) 800, (**c**) 120, and (**d**) 160 features. The results of the methods differ from each other with a various number of features. PC, Pearson correlation; LA, Laplacian score; EN, entropy; MI, mutual information; FI, Fisher score.

**Figure 16:**
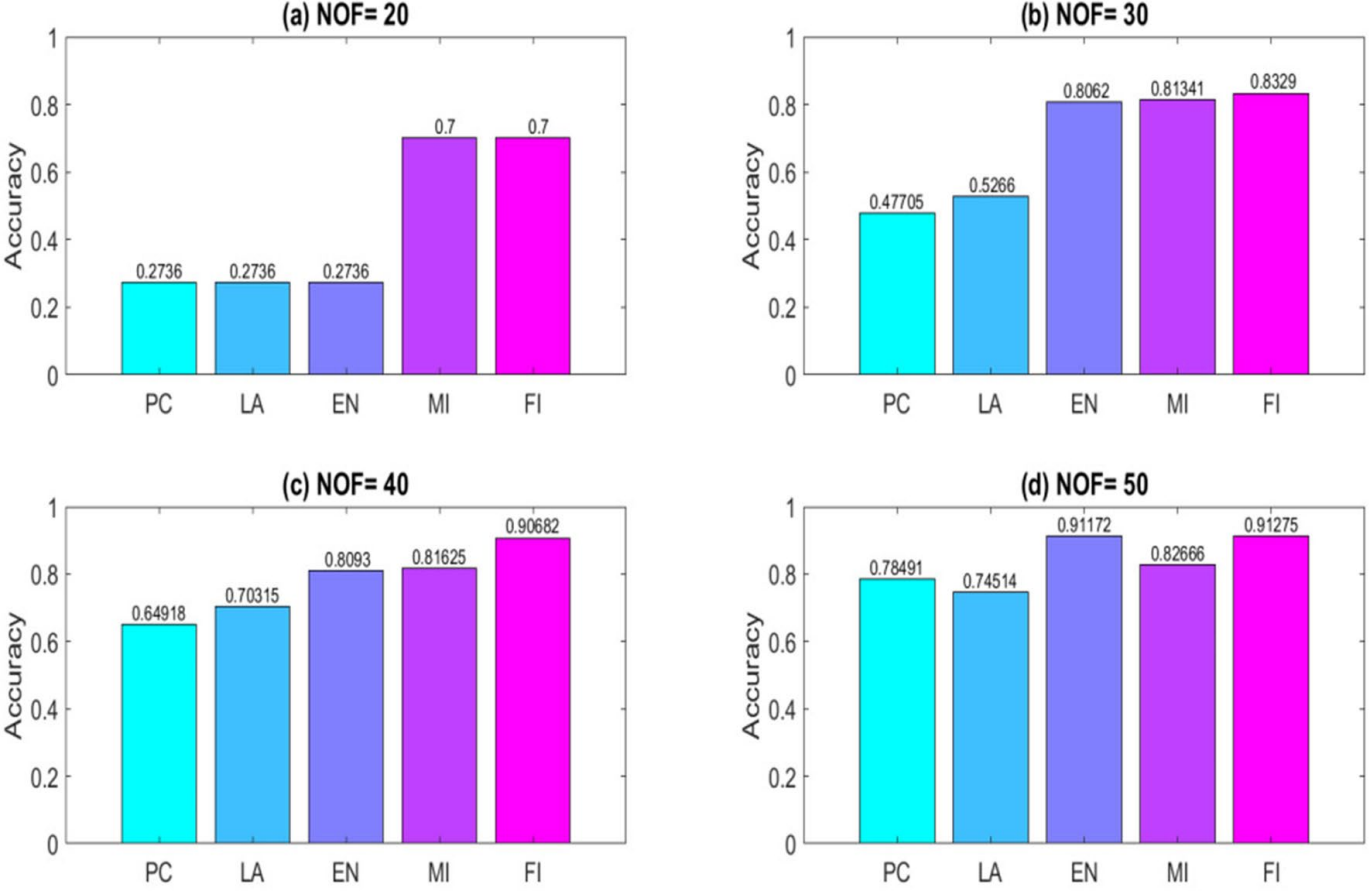
Outcomes of the filter FS methods on the Diabetes dataset with selecting (**a**) 20, (**b**) 30, (**c**) 40, and (**d**) 50 features. The confining total number of features may lead to different results. PC, Pearson correlation; LA, Laplacian score; EN, entropy; MI, mutual information; FI, Fisher score.

**Figure 17:**
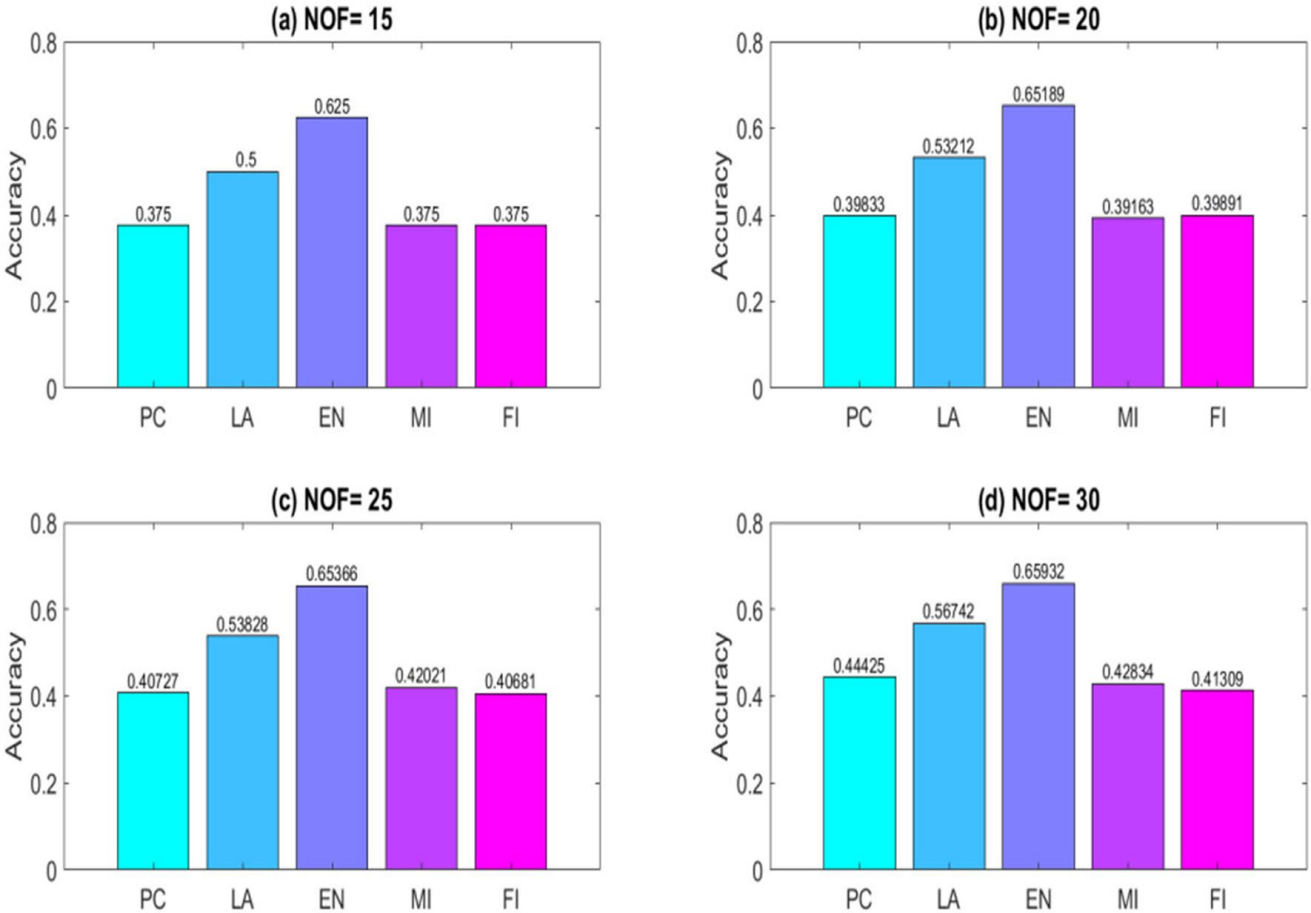
Outcomes of the filter FS methods on the Lung dataset with selecting (**a**) 15, (**b**) 20, (**c**) 25, and (**d**) 30 features. The EN method with an average of 81% accuracy is better than other approaches. PC, Pearson correlation; LA, Laplacian score; EN, entropy; MI, mutual information; FI, Fisher score.

To obtain results, a computer system with a dual-core 2.2 GH processor and 12 GB of RAM was employed. Further, our designed FeatureSelect software application and MATLAB programming language were used for the implementations. In this section, all the obtained outcomes refer to results from the five-fold cross-validation technique. For comparing the algorithms and methods, the same conditions were considered. For example, GA, WCC algorithm, and GA_WCC method allowed to run for an identical time for getting the results. The size of populations for the GA, WCC algorithm, and GA_WCC method was determined using a “trial and error” method and their time-consuming parameter, in which the best performance of the algorithms is observed. Based on the outcomes, the population sizes were considered 100, 20, and 100 for the GA, WCC algorithm, and GA_WCC method, respectively. The mutation and crossover rates were set to 30%, because the GA shows a suitable behavior based on them. In addition to the population size parameters, the WCC algorithm consists of the *match time* (the total number of attempts to change a CS) parameter, which has been set to 2. This parameter was initiated 1 to the GA_WCC method. The outcomes (which encompassed the results of five popular filter FS methods, GA, WCC, a two-step filter-wrapper method (EN_WCC), and the proposed wrapper-wrapper method (GA_WCC)), were divided into the following three categories:

(i) The first category of the results: This class consists of the results obtained from applying the mentioned algorithms and methods to the datasets having more than 50 features and relating to the classification type. Tables 2 and 3 represent the attained outcomes. Also, Fig. 9 depicts the results of the SVM without applying the FS algorithms on the investigated datasets.
  Wrapper-based FS methods improve the performance of SVM, whereas Filter-based FS approaches may reduce its performance. Overall, among the filter methods, the entropy-based (EN) FS method has led to more appropriate results than others. Moreover, between GA and WCC algorithms, WCC yields better outcomes. Hence, a combination of EN and WCC (the so-called EN_WCC) is also investigated and compared against the others. For the Cancer dataset, GA_WCC, GA, and WCC have yielded the best solutions. However, GA_WCC and GA classify the data with six features, whereas WCC classifies them with ten attributes. For the Arrhythmia dataset, the proposed approach outperforms others in terms of the total number of features (NOF) and other classification criteria. For the Diabetes dataset, EN_WCC yielded a minimum number of features and have yielded better outcomes than the filter methods, as observed for the cancer dataset. Nevertheless, the data of GA_WCC, WCC, and GA surpass EN_WCC. Similar outcomes are observed for the other datasets. Tables 2 and 3 show that wrapper and two-step methods are more efficient than the filter ones, and their performance can be sorted as GA_WCC, WCC, GA, and EN_WCC, respectively.
  For further evaluating the methods, receiving operating characteristic (ROC) curves of the methods are shown in Figs. 10 and 11. The area under the curve (AUC) values of the approaches on the datasets of the first class of the outcomes are shown in Table 4. The two-step and wrapper approaches have remarkable functionality compared to the others, and the proposed method outperforms all of them (Figs. 10, 11, Tables 2, 3, and 4). In another evaluation of the algorithm’s performance, the p-value (PV) measurement was considered (Table 5). To this end, every algorithm was performed in 50 individual executions, and the results of the proposed method (GA_WCC) were considered as a test base. Next, the outcomes of the other algorithms were compared with them. Except for the Cancer dataset, in which the effectiveness of the algorithms is the same, the proposed method has outperformed the others for the remaining datasets. Figure 12 also presents boxplots of the algorithms’ outputs obtained using One-Way ANOVA test. Every execution consists of 100 iterations of the algorithms step. At the end of an iteration, the best acquired accuracy was stored, and the convergence behavior of the algorithms were investigated for the datasets including more than 1000 features (Fig. 13). It was observed that the convergence speed of the proposed method is higher than the GA and WCC algorithms (without merging them). As mentioned before, the combined method can efficiently address the limitations of the GA and WCC algorithm (the low convergence of the GA algorithm and the restricted number of CSs in the WCC) and yield better outcomes when combined than when run individually.
  In filter FS methods, determining the total number of features is a challenging problem and plays an essential role in the performance of a model. The results of the five filter approaches are shown in Figs. 14, 15, 16, and 17. These outcomes show the performance of the filter FS methods with a different number of features.
(ii) The second category of results: This section includes the results of the algorithms on the datasets having less than 50 features/attributes. The main goal of this section is to check the effect of FS methods on datasets, which consist of fewer numbers of features. For the small datasets, single wrapper methods do not face special challenges in the FS. Indeed, the mentioned FS methods may obtain the best solution by improving the run time. Hence, in this section, the functionality of the GA and WCC algorithms are inspected. Like for the first part, criteria such as sensitivity, specificity, accuracy, precision, and AUC were investigated. The acquired data are listed in Table 6.
  Without applying the GA and WCC algorithms, SVM alone yields 0.5263, 0.6645, and 0.5812 value of accuracy using the fivefold cross-validation technique on the CHD, SHD, and PID datasets, respectively. By applying the algorithms, the value of accuracy improved for the CHD and SHD datasets and remains unchanged for the PID dataset. Further, the total number of features is remarkably reduced. Thus, the obtained models obtained by applying the algorithms operate faster than the model, which uses all the existing features. Having compared GA and WCC algorithms, WCC was seen to lead to a model with lower number of features and higher values of criteria. Therefore, it might be concluded that the state-of-the-art optimization algorithm can get more acceptable data than others.
(iii) The third category of the results: In this section, the outcomes of the methods and algorithms are evaluated on the regression-based dataset (WDBC and drug datasets). To this end, the criteria such as root mean squared error (RMSE) and the correlation between predicted and real labels were calculated and gathered (Table 7). For the filter FS methods, different numbers of features have been tested, and then, their best results were reported. For the wrapper FS approaches, it is not necessary to limit the total number of features and they can regulate it. Even so, they produce variable results in their different executions, so they must be executed at least 30 times and their best-obtained outcomes among from the executions (different accuracy values of the executions) are reported as a solution to the problem. Thus, several criteria were reported for them, based on the acquired results in 50 individual executions, including confidence interval (CI), p-value, standard deviation (STD), etc.

**Table 2:** Acquired outcomes based on fivefold cross-validation in the first class of results on the Cancer, Arrythmia, Diabetes, and Lung datasets.

Algorithm	NOF	SEN	SPC	PRE	FPR	ACC	F-measure	C-Kappa	dataset
GA	**6**	**1.000**	**1.000**	**1.000**	**0.000**	**1.000**	**1.00**	**0.00**	Cancer
WCC	10	**1.000**	**1.000**	**1.000**	**0.000**	**1.000**	**1.00**	**0.00**	
GA_WCC	**6**	**1.000**	**1.000**	**1.000**	**0.000**	**1.000**	**1.00**	**0.00**	
PC	20	0.958	0.968	0.968	0.032	0.958	0.96	0.06	
LA	20	0.958	0.968	0.968	0.032	0.958	0.96	0.06	
EN	20	0.964	0.968	0.968	0.032	0.964	0.97	0.05	
MI	20	0.961	0.968	0.968	0.032	0.961	0.96	0.05	
FI	20	0.961	0.968	0.968	0.032	0.961	0.96	0.05	
EN_WCC	**6**	0.979	0.980	0.986	0.020	0.979	0.98	0.03	
GA	42	0.721	0.730	0.732	0.270	0.721	0.73	0.37	Arrhythmia
WCC	46	0.786	0.797	0.801	0.203	0.786	0.79	0.29	
GA_WCC	**22**	**0.899**	**0.902**	**0.912**	**0.098**	**0.899**	**0.91**	**0.13**	
PC	40	0.504	0.511	0.530	0.489	0.504	0.52	0.66	
LA	40	0.504	0.511	0.530	0.489	0.504	0.52	0.66	
EN	40	0.509	0.511	0.530	0.489	0.509	0.52	0.65	
MI	40	0.488	0.497	0.427	0.503	0.488	0.46	0.68	
FI	40	0.506	0.511	0.530	0.489	0.506	0.52	0.66	
EN_WCC	67	0.512	0.478	0.511	0.522	0.512	0.51	0.65	
GA	27	0.922	0.815	0.926	0.185	0.922	0.92	0.10	Diabetes
WCC	27	0.949	0.918	0.955	0.082	0.949	0.95	0.07	
GA_WCC	17	**0.993**	**0.998**	**0.995**	**0.002**	**0.993**	**0.99**	**0.01**	
PC	50	0.266	0.726	0.438	0.274	0.266	0.33	0.98	
LA	50	0.271	0.726	0.438	0.274	0.271	0.33	0.97	
EN	50	0.269	0.726	0.438	0.274	0.269	0.33	0.97	
MI	50	0.729	0.300	0.807	0.700	0.729	0.77	0.36	
FI	50	0.727	0.300	0.807	0.700	0.727	0.76	0.36	
EN_WCC	**9**	0.826	0.651	0.828	0.349	0.826	0.83	0.23	
GA	33	0.745	0.929	0.854	0.071	0.745	0.80	0.34	Lung
WCC	38	0.749	0.929	0.854	0.071	0.749	0.80	**0.33**	
GA_WCC	13	**0.749**	**0.917**	**0.875**	**0.083**	**0.749**	**0.81**	**0.33**	
PC	15	0.372	0.625	0.453	0.375	0.372	0.41	0.84	
LA	15	0.492	0.700	0.723	0.300	0.492	0.59	0.68	
EN	15	0.623	0.775	0.813	0.225	0.623	0.71	0.50	
MI	15	0.367	0.625	0.453	0.375	0.367	0.41	0.84	
FI	15	0.373	0.625	0.453	0.375	0.373	0.41	0.84	
EN_WCC	**12**	0.741	0.750	0.830	0.250	0.741	0.78	0.35	

The best outcomes have been highlighted.

NOF, number of features; SEN, sensitivity; SPC, specificity; PRE, precision; FPR, false positive rate; ACC, accuracy; C-Kappa, Cohen’s kappa coefficient; WCC, world competitive contests algorithm; GA, genetic algorithm; PC, Pearson correlation; LA, Laplacian score; EN, entropy; MI, mutual information; FI, Fisher score.

**Table 3:** Acquired outcomes based on fivefold cross-validation in the first class of results on the QSAR, Arcene, MicroMass, and RNA-Seq datasets.

Algorithm	NOF	SEN	SPC	PRE	FPR	ACC	F-measure	C-Kappa	dataset
GA	29	0.778	0.788	0.790	0.212	0.778	0.784	0.296	QSAR
WCC	25	0.848	0.860	0.865	0.140	0.848	0.856	0.202	
GA_WCC	25	**0.970**	**0.974**	**0.984**	**0.026**	**0.970**	**0.977**	**0.040**	
PC	80	0.544	0.552	0.572	0.448	0.544	0.558	0.608	
LA	80	0.544	0.552	0.572	0.448	0.544	0.558	0.608	
EN	80	0.549	0.552	0.572	0.448	0.549	0.560	0.601	
MI	80	0.527	0.536	0.461	0.464	0.527	0.492	0.631	
FI	80	0.546	0.552	0.572	0.448	0.546	0.559	0.605	
EN_WCC	**25**	0.573	0.536	0.572	0.464	0.573	0.572	0.570	
GA	27	0.711	0.720	0.721	0.280	0.661	0.716	0.452	Arcene
WCC	27	0.771	0.781	0.785	0.219	0.721	0.778	0.372	
GA_WCC	**22**	**0.875**	**0.877**	**0.886**	**0.123**	**0.825**	**0.880**	**0.234**	
PC	150	0.512	0.519	0.536	0.481	0.462	0.524	0.717	
LA	150	0.512	0.519	0.536	0.481	0.462	0.524	0.717	
EN	150	0.517	0.519	0.536	0.481	0.467	0.526	0.711	
MI	150	0.498	0.506	0.442	0.494	0.448	0.468	0.737	
FI	150	0.514	0.519	0.536	0.481	0.464	0.525	0.715	
EN_WCC	43	0.540	0.508	0.539	0.492	0.490	0.539	0.681	
GA	16	0.709	0.717	0.719	0.283	0.609	0.714	0.521	MicroMass
WCC	31	0.764	0.774	0.777	0.226	0.664	0.771	0.448	
GA_WCC	26	**0.860**	**0.862**	**0.871**	**0.138**	**0.760**	**0.865**	**0.320**	
PC	50	0.526	0.532	0.548	0.468	0.426	0.537	0.765	
LA	50	0.526	0.532	0.548	0.468	0.426	0.537	0.765	
EN	50	0.530	0.532	0.548	0.468	0.430	0.539	0.760	
MI	50	0.512	0.520	0.461	0.480	0.412	0.485	0.783	
FI	50	0.528	0.532	0.548	0.468	0.428	0.538	0.763	
EN_WCC	**16**	0.553	0.524	0.552	0.476	0.453	0.552	0.730	
GA	48	0.745	0.754	0.755	0.246	0.695	0.750	0.407	RNA-Seq
WCC	51	0.807	0.818	0.822	0.182	0.757	0.815	0.323	
GA_WCC	18	**0.916**	**0.919**	**0.929**	**0.081**	**0.866**	**0.923**	**0.178**	
PC	30	0.536	0.542	0.561	0.458	0.486	0.548	0.686	
LA	30	0.536	0.542	0.561	0.458	0.486	0.548	0.686	
EN	30	0.541	0.542	0.561	0.458	0.491	0.550	0.679	
MI	30	0.520	0.529	0.462	0.471	0.470	0.489	0.706	
FI	30	0.538	0.542	0.561	0.458	0.488	0.549	0.683	
EN_WCC	**17**	0.563	0.531	0.562	0.469	0.513	0.563	0.649	

The best outcomes have been highlighted.

NOF, number of features; SEN, sensitivity; SPC, specificity; PRE, precision; FPR, false positive rate; ACC, accuracy; C-Kappa, Cohen’s kappa coefficient; WCC, world competitive contests algorithm; GA, genetic algorithm; PC, Pearson correlation; LA, Laplacian score; EN, entropy; MI, mutual information; FI, Fisher score.

**Table 4:** The AUC values of the methods in the first category of the results.

dataset	WCC	GA	GA_WCC	PC	LA	EN	MI	FI	EN_WCC
Cancer	**1.000**	**1.000**	**1.000**	0.962	0.962	0.965	0.964	0.964	0.979
Arrhythmia	0.726	0.791	**0.900**	0.507	0.507	0.510	0.507	0.508	0.504
Diabetes	0.868	0.933	**0.995**	0.503	0.501	0.502	0.514	0.513	0.738
Lung	0.837	**0.838**	**0.838**	0.501	0.595	0.698	0.504	0.501	0.745
QSAR	0.783	0.854	**0.972**	0.548	0.548	0.550	0.532	0.549	0.554
Arcene	0.715	0.776	**0.876**	0.515	0.515	0.518	0.502	0.516	0.524
MicroMass	0.713	0.769	**0.861**	0.529	0.529	0.531	0.516	0.530	0.538
RNA-Seq	0.749	0.813	**0.918**	0.539	0.539	0.541	0.525	0.540	0.547

The best outcomes have been highlighted.

WCC, world competitive contests algorithm; GA, genetic algorithm; PC, Pearson correlation; LA, Laplacian score; EN, entropy; MI, mutual information; FI, Fisher score.

**Table 5:** A comparison of the obtained results based on the p-value criterion.

	Cancer	Arrythmia	Diabetes	Lung	QSAR	Arcene	MicroMass	RNA-Seq
GA	1	7.06e−18	1.38e−15	0.001	7.06e−18	7.06e−18	7.06e−18	7.06e−18
WCC	1	7.06e−18	5.41e−13	5.42e−05	7.06e−18	3.31e−20	7.06e−18	7.06e−18

**Table 6:** Results based on fivefold cross-validation in the second class of obtained data.

AL_NAME	NOF	SEN	SPC	PRE	FPR	ACC	AUC	Dataset
GA	**5**	0.676	0.459	0.684	0.541	0.684	0.567	CHD
WCC	8	**0.710**	**0.549**	**0.709**	**0.451**	**0.737**	**0.629**	
GA	24	**0.717**	0.500	**0.717**	0.500	0.710	**0.665**	PID
WCC	**22**	0.707	**0.564**	0.705	**0.436**	**0.716**	**0.665**	
GA	28	0.872	0.854	0.876	0.146	0.873	0.863	SHD
WCC	**6**	**0.887**	**0.887**	**0.891**	**0.113**	**0.891**	**0.887**	

The best outcomes have been highlighted.

NOF, number of features; SEN, sensitivity; SPC, specificity; PRE, precision; FPR, false positive rate; ACC, accuracy; AUC, area under the curve.

**Table 7:** Comparison of the methods on the regression-based datasets.

Dataset	Method	NOF	ET	ER	ER_STD	ER_CI_1	ER_CI_2	ER_PV	ER_TS	CR	CR_STD	CR_CI_1	CR_CI_2	CR_PV	CR_TS
Drug	WCC	44	18.260	**0.080**	0.030	0.128	0.140	3.06043E−21	25.144	**0.962**	0.017	**0.948**	**0.950**	1.07907E−52	312.159
	GA	47	15.021	0.104	0.017	0.129	0.142	5.83777E−28	43.488	0.946	0.012	0.941	0.950	**1.28025E−56**	**426.359**
	PC	49	**4.158**	1.186	–	–	–	–	–	0.329	–	–	–	–	–
	LA	47	4.971	1.182	–	–	–	–	–	0.325	–	–	–	–	–
	EN	50	4.957	0.987	–	–	–	–	–	0.322	–	–	–	–	–
	MI	50	4.485	1.181	–	–	–	–	–	0.322	–	–	–	–	–
	FI	49	4.800	1.186	–	–	–	–	–	0.322	–	–	–	–	–
	EN_WCC	55	10.933	0.491	0.070	0.639	0.691	3.00496E−30	52.271	0.706	0.052	0.548	0.588	6.74362E−32	59.653
	GA_WCC	**24**	17.530	0.105	**0.010**	0.125	**0.133**	**7.9859E−34**	**69.586**	0.884	**0.015**	0.867	0.878	2.42408E−53	328.658
WDBC	WCC	12	23.200	0.030	0.006	0.047	0.051	4.10491E−66	906.110	0.965	0.006	0.948	0.952	6.47489E−29	46.963
	GA	13	35.952	0.034	0.006	0.048	0.054	7.8477E−67	959.312	0.035	0.006	0.940	0.944	1.21143E−27	42.391
	PC	14	**0.414**	0.941	–	–	–	–	–	–	0.326	–	–	–	–
	LA	14	0.442	0.910	–	–	–	–	–	–	0.317	–	–	–	–
	EN	13	0.492	0.949	–	–	–	–	–	–	0.305	–	–	–	–
	MI	14	0.479	0.907	–	–	–	–	–	–	0.294	–	–	–	–
	FI	13	0.496	0.997	–	–	–	–	–	–	0.293	–	–	–	–
	EN_WCC	12	23.200	0.030	0.006	0.047	0.051	4.10491E−66	906.110	0.965	0.006	0.948	0.952	6.47489E−29	46.963
	GA_WCC	**9**	29.242	**0.011**	**0.001**	**0.037**	**0.045**	**6.12101E−71**	**1001.001**	**0.983**	**0.000**	**0.966**	**0.971**	**8.16211E**−**34**	**52.660**

The best outcomes have been highlighted.

WCC, world competitive contests algorithm; GA, genetic algorithm; PC, Pearson correlation; LA, Laplacian score; EN, entropy; MI, mutual information; FI, Fisher score; NOF, number of features; ET, elapsed time; ER, error; STD, standard deviation; CI, confidence interval; TS, test statistics; PV, p-value; CR, correlation; 1, indicates low bound of confidence interval; 2, indicates high bound of confidence interval.

**Figure 18:**
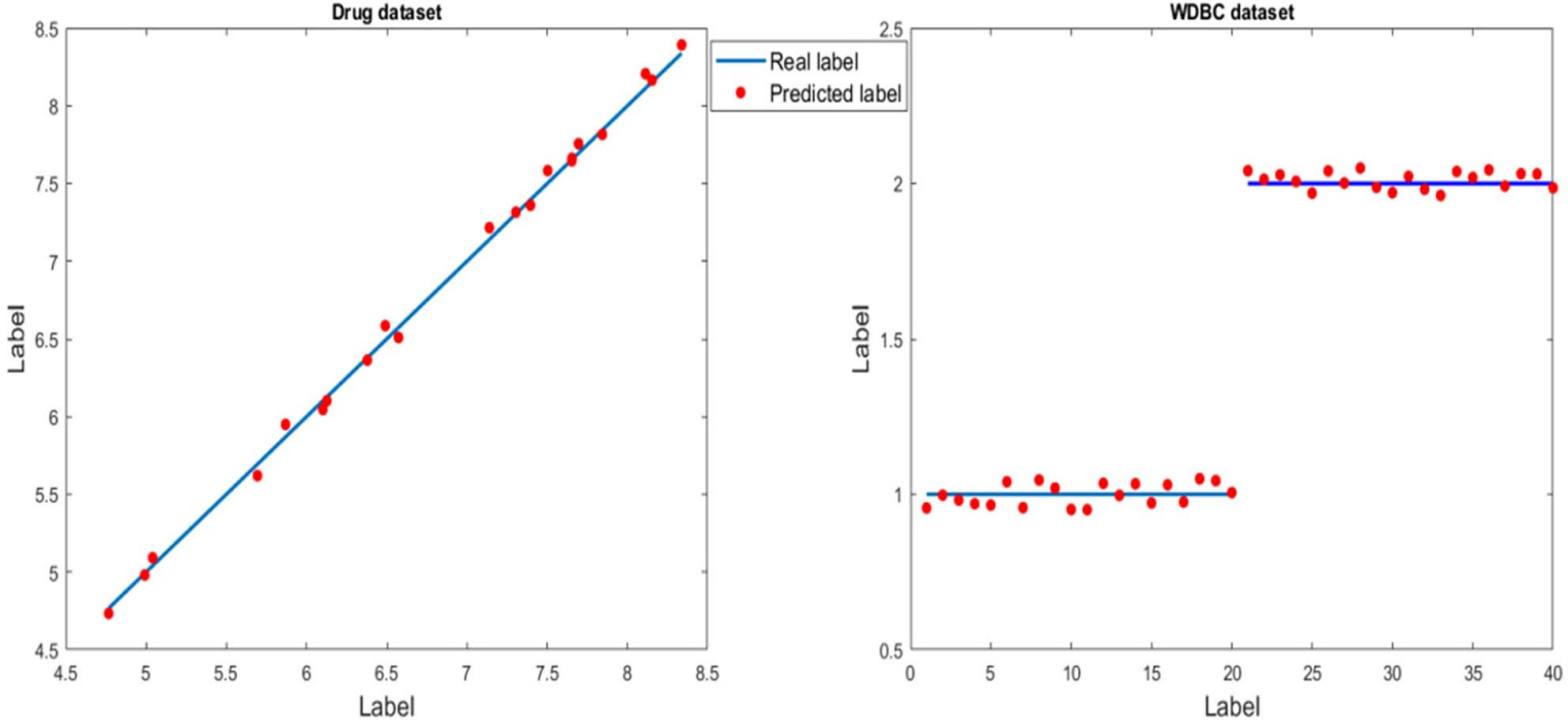
Scatter plot of the proposed method on the Drug and WDBC datasets. Blue and red points indicate real and predicted values, respectively. The value of RMSE for the drug and WDBC datasets are 0.14 and 0.01, respectively.

From the run-time perspective, filter FS methods require less time than wrapper approaches, but do not result in improved outcomes. For instance, for the WDBC dataset, the entropy FS approach yields the minimum value of error and the maximum value of correlation between the predicted and real labels, when the total number of features is limited to 13. The value of correlation can be calculated not only for the entropy method but also for others. As the first class of results, the second one also shows the remarkable performance of the proposed approach (GA_WCC) in terms of error, correlation, the total number of selected features, run-time, etc. Besides, WCC and GA present that wrapper FS method may acquire better results than the filter FS approaches. In Fig. 18, the scatter plots of the proposed method on the regression-based datasets are shown.

## Discussion

Many methods and algorithms have been proposed for selecting an optimal subset of features, which is indeed an NP-hard problem, particularly in machine learning with a biological context. Besides enhancing the separability power of a model, optimal features improve the speed of a model and may lead to valuable results such as acquiring an optimal kit of biomarkers to be used in applications. In this area, it has been shown that two-step FS approaches lead to better outcomes than single methods^59^, and wrapper-based FS methods usually outperform filter and embedded FS techniques^60^. The results of this study also confirm the mentioned observations and allow for the following important key conclusions:

First, wrapper FS methods may obtain an optimal subset of features, which do not require confining the total number of features to a predefined number. Nevertheless, there are some restrictions in determining the total number of selected features. For example, wrapper methods may obtain a subset of attributes with the highest score, while the total number of the selected features may be greater than the required number of features (problem limitations). In this line, we believe that wrapper FS methods are still better than the filter and embedded FS approaches, in large part because they can be formulated in a way to resolve the problem constraints.

Second, limiting the filter methods to a predefined number is a challenging problem and affects the performance of filter FS approaches. The results of this work show that the performance of filter FS approaches vary with the different number of selected features. Thus, this parameter remains a challenge for researchers. However, wrapper methods, which consider a set of features instead of examining each of them separately, do not face this restriction.

Third, the FS is also essential for datasets having a low number of features. In the second part of the results, the performance of wrapper FS methods was investigated on some gold-standard datasets, for which their total number of features is less than 50. Based on other conducted studies^61^, it seems that the FS has been ignored in these works even though it may improve the performance. For this class of datasets, considering the total number of features, single wrapper methods might be a proper method.

Forth, wrapper-wrapper FS methods may be the best option for selecting an optimal subset of features. In the last decade, different types of hybrid methods have been introduced for the FS problem due to their amazing results. However, most of them combine filter-filter or filter-wrapper approaches and a suitable configuration of wrapper-wrapper methods have been ignored. In the present investigation, a wrapper-wrapper approach based on GA and the proposed WCC-algorithm was introduced, which resulted in superior outcomes compared to the other approaches. The WCC algorithm starts with a first population of CSs and, then, applies its operators to them in order to obtain a better solution to the FS problem. The main difference between the WCC algorithm and other optimization algorithms relates to the steps of the algorithm and its operators. The two-step approaches differ from hybrid methods that merge the optimization algorithms such as the whale optimization algorithm and simulated annealing^62^. In this study, to obtain an efficient combination of the algorithms, the advantages and limitations of the GA and WCC algorithm were considered. Since the GA produces various CSs, the WCC algorithm confines them to a limited number. Unlike the WCC algorithm, the GA may suffer from low convergence speed and not show a suitable performance relative to other optimization algorithms. Given the mentioned reasons, GA and WCC algorithm were combined, and the results showed that their combination yields better outcomes.

Fifth, the performance of algorithms and methods varies on different datasets. Every algorithm or method has its own attitude relative to the FS problem, so their functionality may differ on various data. Generally, it is impossible to predict a priori, which of the methods or algorithms is suitable for a given problem. Nonetheless, wrapper-wrapper FS approaches appear promising to produce desired results. As a future work, the proposed method can be applied to other algorithms such as the Salp Swarm Algorithm^63^ and DE^64^ with considering limitations and disadvantages. Also, the proposed method scores a set of features and does not rank the features of the obtained set. To address this limitation, the proposed approach can be combined with state-of-the-art ranking techniques such as SVM-RFE^65,66^.

## Conclusion

For selecting an optimal subset of features, a two-step wrapper-wrapper FS method based on GA and our proposed algorithm (WCC) was introduced and applied to the thirteen biological datasets with different properties. In comparison with other approaches, it can be concluded that two-step techniques may lead to better results than single-step methods. Furthermore, among the two-step approaches, wrapper-wrapper FS methods may be more appropriate than others. For biological applications, it seems that wrapper approaches are the most convenient and reliable method, in large part because they do not need to be restricted to a predefined number of features. Taken together, based on our findings, wrapper-wrapper FS methods can be used to optimize the FS problems and result in robust and desired outcomes.
